# Preoperative Left Ventricular Thrombus and Midterm Outcomes Following Left Ventricular Assist Device Support

**DOI:** 10.3390/jcm15093322

**Published:** 2026-04-27

**Authors:** Umit Kahraman, Berk Dacik, Sedat Karaca, Ahmet Daylan, Serkan Ertugay, Emrah Oguz, Sanem Nalbantgil, Cagatay Engin, Mustafa Ozbaran, Tahir Yagdi

**Affiliations:** 1 Department of Cardiovascular Surgery, Faculty of Medicine, Ege University, 35100 Izmir, Turkey; umitkahraman81@gmail.com (U.K.); berk.dacik@ege.edu.tr (B.D.); ahmet.daylan@ege.edu.tr (A.D.); serkan.ertugay@ege.edu.tr (S.E.); emrah.oguz@ege.edu.tr (E.O.); cagatay.engin@ege.edu.tr (C.E.); mustafa.ozbaran@ege.edu.tr (M.O.); 2 Department of Cardiovascular Surgery, Sanliurfa Training and Research Hospital, 63250 Sanliurfa, Turkey; karacasedat34@gmail.com; 3 Department of Cardiology, Faculty of Medicine, Ege University, 35100 Izmir, Turkey; sanem.nalbantgil@ege.edu.tr

**Keywords:** left ventricular thrombus, left ventricular assist device, thromboembolic complications

## Abstract

**Background:** Preoperative left ventricular thrombus (LVT) may complicate left ventricular assist device (LVAD) implantation by increasing surgical complexity and potentially predisposing patients to thromboembolic events. However, the clinical significance of LVT in the era of LVAD support remains unclear. This study aimed to evaluate whether preoperative LVT influences early and mid-term outcomes after durable LVAD implantation. **Methods:** This retrospective, single-center cohort study included 81 adult patients who underwent LVAD implantation between January 2012 and December 2024. Twenty-one patients had documented preoperative LVT. Propensity score matching (1:1) was performed, resulting in 21 matched pairs. All patients with LVT underwent intraoperative thrombectomy. Postoperative outcomes included ischemic and hemorrhagic stroke, pump thrombosis, infectious complications, ventricular arrhythmias, duration of device support, and postoperative echocardiographic parameters. **Results:** After matching, baseline characteristics were generally comparable. Ischemic stroke incidence was identical (4.8% vs. 4.8%). Hemorrhagic stroke occurred in one LVT patient (4.8%) and in none of the controls. Pump thrombosis was numerically higher in LVT patients (9.5% vs. 4.8%) but not statistically significant. Rates of driveline infection (33.3% vs. 57.1%), bloodstream infection (19.0% vs. 28.6%), and ventricular arrhythmias (19.0% vs. 23.8%) were similar. Postoperative echocardiographic parameters and aortic valve opening patterns were comparable. The median LVAD support duration did not differ significantly (1003 vs. 821 days). **Conclusions:** Preoperative LVT was not associated with statistically significant differences in adverse outcomes following LVAD implantation when managed with surgical thrombectomy and standardized anticoagulation. However, given the small matched cohort and wide confidence intervals, clinically meaningful differences cannot be excluded. These findings require confirmation in larger prospective studies.

## 1. Introduction

Advanced heart failure (HF) remains a major cause of morbidity and mortality worldwide despite substantial advances in pharmacological and device-based therapies. For patients with end-stage HF who remain refractory to optimal medical management, durable left ventricular assist device (LVAD) implantation has become an established therapeutic option, serving either as a bridge to transplantation or as destination therapy. The introduction of continuous-flow LVADs has markedly improved survival and reduced device-related complications compared with earlier pulsatile devices. In the contemporary era, nearly all LVAD implantations involve continuous-flow devices, accounting for approximately 95% of implants. Registry data, including the Interagency Registry for Mechanically Assisted Circulatory Support (INTERMACS), have demonstrated significant improvements in outcomes with current-generation devices, with overall survival approaching 80% at 1 year and 70% at 2 years [1,2]. In the most recent era, 1- and 2-year survival have improved compared with the period between 2010 and 2014 (82.3% and 73.1% vs. 80.5% and 69.1%, respectively; *p* < 0.0001), while the incidence of stroke has declined to approximately 12.7% at 1 year [3]. Despite these advances, major bleeding and infection remain the most common adverse events, and heart failure progression and multisystem organ failure continue to represent the leading causes of death following LVAD implantation [4].

Left ventricular thrombus (LVT) is a well-recognized complication in patients with severe left ventricular systolic dysfunction. In the contemporary era, advanced HF represents the most common clinical substrate for LVT formation, particularly in patients with marked ventricular dilation and severely reduced ejection fraction [5]. Thrombus formation in this setting reflects the combined effects of blood stasis, endothelial injury, and hypercoagulability. In addition to HF-related cardiomyopathy, extensive myocardial injury—most commonly following anterior myocardial infarction—remains an important precipitating factor. The presence of LVT carries significant clinical implications, as it increases the risk of systemic thromboembolism and ischemic stroke [6]. Among patients with systolic dysfunction, the prevalence of LVT detected by delayed-enhancement cardiac magnetic resonance imaging (CMR) has been reported to be approximately 7%, consistent with the 7–10% prevalence range described in similar CMR-based cohorts [7,8].

The presence of LVT in patients undergoing LVAD implantation poses several theoretical and practical challenges. Intraoperative thrombus removal may increase surgical complexity and cardiopulmonary bypass duration, while residual thrombotic material raises concern for early postoperative embolic complications or device thrombosis. Although modern continuous-flow LVADs are designed to optimize flow dynamics and minimize areas of blood stasis, patients remain at inherent risk for thromboembolic events and therefore require lifelong anticoagulation [9].

Several retrospective studies have reported a potential association between pre-existing intracardiac thrombus and adverse postoperative outcomes, including early stroke and mortality, following LVAD implantation [10,11]. However, these findings are not consistent across studies, and the overall strength of this association remains uncertain. The proportion of new-generation devices did not exceed 30%, whereas our study predominantly included new-generation devices known to have better results. This situation likely minimized the impact of device-related adverse events on our findings, although the independent significance of thrombus presence remains difficult to ascertain given the limited sample size. The prognostic significance of pre-existing intracardiac thrombus remains incompletely defined in the contemporary LVAD setting. In this study, we evaluated this issue using a propensity score-matched design in a cohort of patients undergoing durable LVAD implantation, with surgical thrombectomy performed.

Recent advances in left ventricular assist device (LVAD) therapy have been accompanied not only by improvements in device technology but also by refinements in surgical techniques and perioperative management strategies. Newer-generation continuous-flow systems have demonstrated improved hemocompatibility and reduced device-related complications compared with earlier devices [2]. In parallel, advances in operative experience and postoperative management, including optimized anticoagulation and supportive medical therapy, have contributed to improved overall outcomes. Contemporary registry data also indicate a decline in major complications such as stroke, which has decreased to approximately 12.7% at 1 year in the modern era [3]. Collectively, these developments reflect a broader evolution in the management of patients undergoing LVAD implantation.

Against this evolving clinical background, the impact of preoperative LVT remains incompletely defined. Therefore, the present study aimed to evaluate the clinical impact of preoperative left ventricular thrombus in patients undergoing durable LVAD implantation. Specifically, we compared thromboembolic complications, device-related adverse events, infectious outcomes, and postoperative echocardiographic parameters between patients with and without preoperative LVT to determine whether its presence independently influences early and mid-term outcomes following LVAD support.

## 2. Materials and Methods

### 2.1. Study Design and Population

This single-center retrospective observational cohort study was conducted at the Dept. of Cardiovascular Surgery of Ege University and included 81 adult patients who underwent continuous-flow left ventricular assist device (LVAD) implantation between January 2012 and December 2024.

The study protocol was approved by the Ege University Ethics Committee (approval number: 26-2.1T/76), and all procedures were conducted in accordance with the principles of the Declaration of Helsinki.

### 2.2. Definition and Assessment of Left Ventricular Thrombus

All patients underwent preoperative transthoracic and transesophageal echocardiographic evaluation for the assessment of left ventricular thrombus (LVT). LVT was defined as a distinct echodense mass within the left ventricular cavity, adjacent to but acoustically distinct from the endocardium, and visible in at least two orthogonal imaging planes.

Intraoperatively, transesophageal echocardiography was routinely used to guide optimal apical inflow cannula positioning. Following apical incision for device implantation, the left ventricular cavity was directly inspected, allowing confirmation of thrombus presence and the performance of surgical thrombectomy. No discrepancies were observed between preoperative imaging and intraoperative findings in the present cohort.

### 2.3. Study Groups and Inclusion Criteria

The initial study population consisted of patients with documented preoperative LVT and a contemporaneous control group without LVT who underwent LVAD implantation during the same study period.

Patients with incomplete clinical or echocardiographic data were excluded from the analysis.

### 2.4. Data Collection and Baseline Variables

Demographic, clinical, laboratory, echocardiographic, operative, and follow-up data were retrieved from institutional electronic medical records.

Baseline variables included age, sex, body mass index (BMI), cardiomyopathy etiology (ischemic cardiomyopathy, dilated cardiomyopathy, arrhythmogenic right ventricular dysplasia, or restrictive cardiomyopathy), hypertension, diabetes mellitus, preoperative atrial fibrillation, carotid artery disease, and a history of cerebrovascular events. Lipid profile parameters, including low-density lipoprotein (LDL) levels, were also recorded.

### 2.5. Echocardiographic Assessment

Preoperative echocardiographic parameters included left ventricular end-diastolic diameter (LVEDD), left ventricular end-systolic diameter (LVESD), and tricuspid annular plane systolic excursion (TAPSE).

### 2.6. Surgical Procedure and Postoperative Management

All LVAD implantations were performed via standard median sternotomy under cardiopulmonary bypass. In all patients with preoperatively identified LVT (*n* = 21), surgical thrombectomy was performed prior to apical cannula placement. Complete removal of thrombotic material was achieved in all cases, and thorough intraoperative inspection of the left ventricular cavity was performed to ensure the absence of residual thrombus. No residual thrombus was detected on intraoperative assessment or postoperative echocardiographic evaluation.

All patients with echocardiographically detected LVT underwent intraoperative inspection during LVAD implantation, and only cases with visually confirmed thrombus by the operating surgeon were included in the LVT cohort.

### 2.7. Preoperative and Postoperative Anticoagulation Management

Preoperative anticoagulation management was standardized across the study cohort. Among patients with atrial fibrillation, oral anticoagulation (vitamin K antagonist or direct oral anticoagulant) was discontinued prior to surgery and bridged with subcutaneous enoxaparin. Patients in sinus rhythm with documented left ventricular thrombus were also initiated on enoxaparin therapy upon hospital admission. In all cases, enoxaparin was discontinued 12 h before the scheduled operation. Precise data on the total duration of preoperative anticoagulation were not available, as therapy was initiated at the time of hospital admission and its duration varied depending on the timing of surgery.

In the postoperative period, anticoagulation therapy was initiated according to the institutional protocol. After adequate hemostasis was achieved, intravenous unfractionated heparin was started, followed by oral vitamin K antagonist therapy targeting an international normalized ratio (INR) range of 2.0–3.0, adjusted according to individual thromboembolic and hemorrhagic risk profiles.

Anticoagulation control was assessed using the time in therapeutic range (TTR), which was high in both groups, indicating effective and stable anticoagulation during follow-up. The median TTR was 80.0% (IQR, 80.0–100.0; range, 60–100) in the left ventricular thrombus group (*n* = 21) and 80.0% (IQR, 78.8–80.0; range, 50–100) in the non-thrombus group (*n* = 20).

### 2.8. Postoperative Outcome Assessment

Postoperative outcomes assessed during follow-up included ischemic stroke, hemorrhagic stroke, pump thrombosis, driveline infection, bloodstream infection, ventricular arrhythmias (ventricular fibrillation or ventricular tachycardia), left atrial thrombus formation, aortic valve opening pattern, duration of device support, postoperative echocardiographic parameters (LVEDD, LVESD, TAPSE), and amiodarone use.

### 2.9. Definitions

Clinical outcomes were defined in accordance with commonly accepted criteria, including alignment with INTERMACS and ISHLT recommendations where applicable.

Ischemic stroke was defined as a new, persistent (>24 h) focal neurological deficit of presumed vascular origin, confirmed by neuroimaging (computed tomography or magnetic resonance imaging) demonstrating cerebral infarction. Hemorrhagic stroke was defined as any intracranial hemorrhage confirmed by imaging, irrespective of associated neurological symptoms.

Pump thrombosis was defined according to standard mechanical circulatory support criteria, based on a combination of clinical, laboratory, and device-related findings, including evidence of hemolysis (elevated lactate dehydrogenase), increased pump power consumption, abnormal pump parameters, and/or the need for therapeutic intervention such as thrombolysis or pump exchange.

Driveline infection was defined as a localized infection at the driveline exit site characterized by erythema, warmth, tenderness, or purulent discharge, with or without positive microbiological cultures, requiring antimicrobial therapy. Bloodstream infection was defined as the presence of a positive blood culture in conjunction with clinical signs of systemic infection requiring targeted antibiotic treatment.

Ventricular arrhythmias were defined as sustained ventricular tachycardia or ventricular fibrillation requiring pharmacological treatment, electrical cardioversion/defibrillation, or device-based therapy.

### 2.10. Statistical Analyses

The statistical analyses were performed using IBM Statistical Package for the Social Sciences (SPSS) for macOS, version 30.0 (IBM Corp., Armonk, NY, USA). Categorical variables are presented as numbers and percentages (*n* (%)), while continuous variables are reported as medians (interquartile range, IQR).

To reduce baseline imbalance between patients with left ventricular thrombus (LVT) and the control group, propensity score matching (PSM) was applied. The initial cohort consisted of 21 patients with LVT and 60 control patients. Propensity scores were estimated using a logistic regression model with LVT status as the dependent variable. Clinically relevant and potential confounding variables, including age, preoperative atrial fibrillation, preoperative carotid artery disease, and preoperative cerebrovascular accident (CVA), were included in the model.

Matching was performed at a 1:1 ratio without replacement using an optimal matching algorithm based on the estimated propensity scores. As optimal matching was applied, no additional caliper restriction was used. Matching was restricted to patients within the region of common support, resulting in 21 matched pairs consisting of 21 patients with LVT and 21 control patients.

Covariate balance after matching was assessed using standardized mean differences (SMDs). Absolute SMD values <0.10 were considered indicative of good balance, values between 0.10 and 0.20 were considered acceptable, and values >0.20 were interpreted as residual imbalance. Variables included in the propensity model were selected based on clinical relevance and previously reported risk factors for thromboembolic events in LVAD patients. Notably, no female patients were present in the control group, which limited complete sex matching in the propensity score analysis. In addition, residual imbalance remained for sex and hypertension, reflecting the limitations imposed by the small sample size and baseline population characteristics.

Since preoperative CVA showed no variability within the cohort, this variable did not contribute to the matching process in practice.

In the matched cohort, group comparisons were performed taking the matched data structure into account. Categorical variables were compared using McNemar’s test (and the exact McNemar test when appropriate), while continuous variables were analyzed using the Wilcoxon signed-rank test. Effect sizes were reported as matched odds ratios (with 95% confidence intervals) for categorical variables and as paired median differences (patient − control, percentile range) for continuous variables.

A two-sided *p*-value < 0.05 was considered statistically significant in all analyses.

## 3. Results

### 3.1. Study Population and Matching

A total of 81 patients underwent durable LVAD implantation during the study period and met the inclusion criteria. Among them, 21 patients had documented preoperative left ventricular thrombus (LVT), while 60 patients did not. After 1:1 propensity score matching, 21 patients with LVT and 21 control patients without thrombus were included in the analysis.

### 3.2. Baseline Characteristics

Baseline demographic and clinical characteristics were generally comparable between groups, although residual imbalance remained for certain variables. All patients in the control group were male (100%), whereas 81% of patients in the thrombus group were male and 19% were female (|SMD| = 0.649). The median age was comparable between groups (56 (47–62) vs. 57 (49–62) years; |SMD| = 0.175). Median BMI was slightly lower in the thrombus group (23.4 (21.2–24.8) vs. 25 (23–26.7) kg/m^2^; |SMD| = 0.296). Regarding cardiomyopathy etiology, ischemic cardiomyopathy was present in 57.1% of controls and 52.4% of thrombus patients (|SMD| = 0.096), while dilated cardiomyopathy was observed in 38.1% of both groups (|SMD| = 0.000). One patient (4.8%) in the thrombus group had arrhythmogenic right ventricular dysplasia, and none had restrictive cardiomyopathy. Hypertension was more frequent in the thrombus group (61.9% vs. 33.3%; |SMD| = 0.572), as was diabetes mellitus (33.3% vs. 19%; |SMD| = 0.325). Preoperative atrial fibrillation was present in 28.6% of thrombus patients and 19% of controls (|SMD| = 0.224). Among patients with atrial fibrillation, preoperative anticoagulation therapy consisted of rivaroxaban (*n* = 3, 14.3%), edoxaban (*n* = 2, 9.5%), and warfarin (*n* = 1, 4.8%) in the LVT group, and edoxaban (n = 2, 9.5%), rivaroxaban (*n* = 1, 4.8%), and apixaban (*n* = 1, 4.8%) in the non-LVT group. These agents were discontinued prior to surgery and bridged with subcutaneous enoxaparin. Patients in sinus rhythm with documented LVT were also initiated on enoxaparin upon hospital admission. In all cases, enoxaparin was discontinued 12 h before the scheduled operation. One control patient (4.8%) had a history of cerebrovascular accident, while no patients in the thrombus group had prior events (|SMD| = 0.312). No patients in either group had carotid artery disease. Preoperative LDL levels were similar (75 (60–103) vs. 83 (69–98) mg/dL; |SMD| = 0.141) (Table 1).

### 3.3. Preoperative Echocardiographic and Device Characteristics

Preoperative echocardiographic and device characteristics were largely comparable. Median LVEDD was 67 mm (60–70) in controls versus 67 mm (65–71) in the thrombus group (|SMD| = 0.558), and median LVESD was 61 mm (56–66) versus 60 mm (57–69), respectively (|SMD| = 0.255). TAPSE values were 13 mm (11–16) in controls and 14 mm (11–17) in thrombus patients (|SMD| = 0.186). HeartMate 3 (Abbott Inc., Abbott Park, IL, USA) was the most frequently implanted device (85.7% in controls vs. 71.4% in the thrombus group; |SMD| = 0.348), followed by HeartWare (HVAD) (Medtronic, Inc., Minneapolis, MN, USA) (14.3% vs. 19%; |SMD| = 0.130). HeartMate II (Abbott Inc., Abbott Park, IL, USA) was used only in the thrombus group (9.5%; |SMD| = 0.447). One patient (4.8%) in the thrombus group had a prior mechanical mitral valve replacement, whereas none in the control group did (|SMD| = 0.312). Overall, baseline characteristics were reasonably balanced after matching, although residual imbalance remained for sex distribution and hypertension (|SMD| > 0.20) (Table 1).

### 3.4. Postoperative Outcomes

Postoperative outcomes did not differ significantly between groups. In the matched cohort, ischemic stroke occurred at identical rates (4.8% (1/21) vs. 4.8% (1/21); *p* = 1.000). Hemorrhagic stroke was observed in one LVT patient (4.8%) and none in controls (*p* = 1.000). Pump thrombosis occurred numerically more frequently in LVT patients (9.5% (2/21) vs. 4.8% (1/21); *p* = 1.000), though this study was underpowered to detect this difference. Left atrial thrombus formation was observed in one LVT patient only (*p* = 1.000). Ventricular arrhythmias (ventricular fibrillation or tachycardia) were observed in 19% (4/21) of LVT patients and 23.8% (5/21) of controls (*p* = 1.000). Amiodarone use was slightly more frequent in the thrombus group (52.4% (11/21) vs. 33.3% (7/21); *p* = 0.289) (Table 2).

Infectious complications were common in both groups. Driveline infections occurred in 33.3% (7/21) of LVT patients and 57.1% (12/21) of controls (*p* = 0.227), whereas bloodstream infections were observed in 19% (4/21) versus 28.6% (6/21), respectively (*p* = 0.688). The median duration of device support was 1003 days (427–1935) in the thrombus group and 821 days (709–1028) in controls (*p* = 0.892) (Table 2).

Postoperative echocardiographic parameters and aortic valve opening patterns were also similar. Median LVEDD was 68 mm (63–72) in LVT patients versus 65 mm (53–69) in controls (*p* = 0.103), and median LVESD was 59 mm (55–67) versus 53 mm (46–65) (*p* = 0.096). TAPSE values were identical in both groups (14 (12–16) mm; *p* = 0.592). Continuous or intermittent aortic valve opening was observed in 61.9% of LVT patients and 81% of controls, whereas persistent closure occurred in 38.1% and 19%, respectively (*p* = 0.289) (Table 2).

In this propensity score-matched analysis, no statistically significant differences were observed between patients with and without preoperative LVT in terms of thromboembolic events, pump thrombosis, infectious complications, or adverse echocardiographic remodeling during early and mid-term follow-up after LVAD implantation with surgical thrombectomy.

## 4. Discussion

In this propensity score-matched analysis, we observed no statistically significant differences in the rates of thromboembolic complications, pump thrombosis, or infectious events between patients with and without preoperative left ventricular thrombus (LVT) following continuous-flow left ventricular assist device (CF-LVAD) implantation under meticulous surgical thrombectomy and standardized anticoagulation protocols. However, given the limited sample size and low number of events, these findings should be interpreted with caution, as the study may be underpowered to detect clinically meaningful differences. Furthermore, the very wide confidence intervals observed in the effect size estimates—most notably a matched odds ratio of 2.00 (95% CI: 0.18–22.1) for pump thrombosis—reflect substantial statistical uncertainty, underscoring the limited power of the present analysis. Therefore, the potential impact of preoperative LVT on outcomes warrants further investigation in larger cohorts.

Patients with preoperative LVT exhibited a modestly lower median body mass index compared with controls, though this difference is unlikely to be clinically meaningful. The distribution of underlying cardiomyopathy etiologies was comparable between groups, with ischemic and dilated cardiomyopathy predominating. These observations suggest that thrombus formation may be more closely related to the severity of ventricular dysfunction rather than to a specific cardiomyopathy subtype. The higher prevalence of hypertension and diabetes mellitus in the LVT group aligns with established prothrombotic mechanisms involving endothelial dysfunction, systemic inflammation, and altered hemostatic profiles. Notably, comparable preoperative low-density lipoprotein levels between groups suggest that lipid-related risk factors may not have substantially influenced thrombus formation in this cohort. Collectively, these findings support the concept that LVT development in advanced heart failure is likely multifactorial, reflecting the interplay among hemodynamic stasis, myocardial injury, and systemic comorbidities. Postoperative ischemic stroke occurred at identical rates in both groups (4.8% vs. 4.8%, *p* = 1.000), despite a numerical imbalance in preoperative cerebrovascular accident history. Hemorrhagic stroke was observed exclusively in the LVT group (4.8%), though this difference did not achieve statistical significance. These outcomes are noteworthy given that stroke remains a major determinant of morbidity and mortality in LVAD recipients. Large registry data suggest that stroke continues to be clinically relevant among LVAD recipients, with an incidence rate of 0.123 strokes per patient-year; ischemic and hemorrhagic strokes accounted for 51.4% and 48.6%, respectively [12]. Technological advances have substantially improved neurological outcomes; the MOMENTUM 3 trial demonstrated significantly higher survival free from disabling stroke or device replacement with the fully magnetically levitated centrifugal-flow HeartMate 3 compared with the axial-flow HeartMate II (76.9% vs. 64.8% at 2 years, *p* < 0.001) [2]. Contemporary analyses further support device-specific variation in neurological event rates, with HeartMate 3 (Abbott Inc., Abbott Park, IL, USA) generally demonstrating lower-events-per-patient-year rates than HeartMate II (Abbott Inc., Abbott Park, IL, USA) and HVAD (Medtronic, Inc., Minneapolis, MN, USA) (all neurologic events: HM3 0.17–0.21; HMII 0.19–0.26; HVAD (Medtronic, Inc., Minneapolis, MN, USA) 0.16–0.28) [13].

In our cohort, the predominance of newer-generation centrifugal-flow devices (85.7% in controls vs. 71.4% in LVT), combined with systematic intraoperative thrombectomy, likely contributed to the absence of excess ischemic stroke risk. In some of the previous studies mentioned above [10,11], the proportion of new-generation devices did not exceed 30%, whereas our study predominantly included new-generation devices known to have better results. This situation likely minimized the impact of device-related adverse events on our findings, although the independent significance of thrombus presence remains difficult to ascertain given the limited sample size.

The isolated hemorrhagic stroke in the LVT group may reflect the inherent challenges of anticoagulation management in mechanical circulatory support rather than direct consequences of pre-existing thrombus. These findings suggest that both device selection and surgical technique may be important determinants of postoperative embolic risk.

Pump thrombosis occurred numerically more frequently in LVT patients (9.5% vs. 4.8%), though this difference was not statistically significant. This complication carries substantial clinical significance given its association with hemolysis, device malfunction, and mortality. Historical data from HeartMate II recipients demonstrated a marked increase in pump thrombosis incidence after 2011, with 180-day mortality reaching 35.6% following thrombosis diagnosis compared with 16.8% in those without thrombosis [14]. However, the MOMENTUM 3 trial reported dramatically lower device exchange rates with HeartMate 3 (Abbott Inc., Abbott Park, IL, USA) compared with HeartMate II (Abbott Inc., Abbott Park, IL, USA) (1.6% vs. 17.0%, hazard ratio 0.08, 95% confidence interval 0.03–0.27, *p* < 0.001), most exchanges in the axial-flow group being attributable to pump thrombosis or severe hemolysis [2].

Despite technological advances, pre-existing intracavitary thrombus remains a fundamental surgical challenge. Complete thrombus extraction, precise inflow cannula positioning, and meticulous ventricular cavity inspection are essential technical elements. In this regard, Dogan et al. reported that LVAD implantation combined with systematic intraoperative thrombectomy in patients with left ventricular thrombus is feasible and can be performed safely without excess early embolic complications [15]. Our findings suggest that comprehensive intraoperative thrombus clearance—rather than device selection alone—may be an important modifiable factor mitigating thromboembolic risk in this population. Thus, while centrifugal-flow technology enhances hemocompatibility, surgical precision may remain a key contributor to postoperative outcomes.

Infectious complications were common in both groups, consistent with contemporary LVAD experience. Driveline infection occurred in 33.3% of LVT patients and 57.1% of controls, while bloodstream infection rates were 19.0% and 28.6%, respectively, without statistically significant differences. Infection remains one of the leading causes of morbidity following durable LVAD implantation. In contemporary registry analyses, major infection has been identified as the most frequent adverse event, with freedom from major infection at 1 year observed in only 60% of patients supported with axial-flow devices, 57% of those with hybrid-levitation centrifugal-flow devices, and 67% of patients receiving fully magnetically levitated centrifugal-flow platforms [16]. Data from the Interagency Registry for Mechanically Assisted Circulatory Support annual reports further indicate that infection—particularly driveline infection—remains among the most frequent adverse events during long-term mechanical circulatory support and contributes substantially to rehospitalization and late mortality. Importantly, preoperative LVT was not associated with a statistically significant increase in infectious risk in patients managed with surgical thrombectomy, although this finding should be interpreted with caution given the limited sample size. This observation supports the concept that LVT may primarily represent a thrombotic substrate rather than a systemic inflammatory or infective risk modifier.

Preoperative atrial fibrillation was numerically more frequent in the LVT group (28.6% vs. 19.0%), though this imbalance remained within acceptable limits after propensity score matching. Postoperative ventricular arrhythmias occurred at comparable rates (19.0% vs. 23.8%, *p* = 1.000), and amiodarone use was more frequent in the LVT group (52.4% vs. 33.3%), without statistical significance (*p* = 0.289). Both atrial and ventricular arrhythmias may influence thromboembolic risk in patients with advanced heart failure supported with LVAD therapy, albeit through distinct mechanisms. Atrial fibrillation is a well-established prothrombotic condition characterized by atrial mechanical dysfunction, blood stasis, and endothelial activation. In a cohort of LVAD recipients, preoperative atrial fibrillation was independently associated with a significantly lower freedom from thromboembolic events at 1 and 2 years compared with patients without atrial fibrillation (62% and 46% vs. 79% and 72%, respectively; *p* < 0.001) [17]. Current stroke prevention guidelines therefore recommend therapeutic anticoagulation in patients with atrial fibrillation [18].

However, under LVAD support—where structured anticoagulation is routinely maintained and left ventricular unloading reduces intracavitary stasis—the incremental thromboembolic contribution of atrial fibrillation may be attenuated compared with non-supported heart failure populations. Ventricular arrhythmias, in contrast, are not traditionally regarded as primary drivers of thrombus formation. Rather, accumulating evidence suggests that post-LVAD ventricular arrhythmias largely reflect the burden of underlying structural myocardial disease and pre-existing arrhythmic substrate. In continuous-flow LVAD recipients, Garan AR and colleagues reported that pre-operative ventricular arrhythmia were the strongest predictor of post-operative arrhythmia, whereas absence of pre-operative ventricular arrhythmia conferred a markedly lower risk (4.0% vs. 45.5%; *p* < 0.001) [19].

In this context, the comparable rates of postoperative ventricular arrhythmias observed in our cohort suggest that preoperative LVT was not associated with an independent arrhythmogenic effect or a statistically significant increase in subsequent thromboembolic events when meticulous surgical thrombectomy and standardized anticoagulation protocols are applied. Although atrial fibrillation was numerically more frequent in the LVT group, this did not appear to translate into excess postoperative stroke or pump thrombosis. Collectively, these findings should be interpreted with caution and suggest that under contemporary LVAD therapy, thromboembolic risk may be more closely related to effective ventricular unloading, device hemocompatibility, therapeutic anticoagulation, and surgical thrombus clearance than to the presence of atrial or ventricular arrhythmias alone.

The median duration of LVAD support was comparable between groups (1003 days vs. 821 days, *p* = 0.892), indicating that patients with preoperative LVT were not selectively censored by shorter follow-up that might otherwise mask late complications. Long-term real-world registry data with fully magnetically levitated centrifugal-flow devices suggest sustained survival beyond 2 years, with 2-year survival rates exceeding 80% and acceptable adverse event profiles. For example, the multinational ELEVATE registry reported 2-year survival of approximately 83.4% in HeartMate 3 recipients, while 5-year survival remained over 60%, with sustained improvements in functional capacity and relatively low hemocompatibility-related complications [20].

Within this contemporary framework of improving long-term outcomes, preoperative LVT was not associated with a statistically significant increase in mid-term support failure in patients managed with meticulous surgical thrombectomy and standardized therapeutic anticoagulation, although this finding should be interpreted with caution given the limited sample size. The apparent absence of a significant effect may extend beyond the early postoperative phase, although larger studies are needed to confirm this observation. Collectively, these findings suggest that effective ventricular unloading, optimized device hemocompatibility, and comprehensive perioperative management may be important determinants of favorable long-term LVAD outcomes. Preoperative echocardiographic parameters were comparable between groups. Median left ventricular end-diastolic diameter was 67 mm in both groups (interquartile range: 60–70 mm vs. 65–71 mm; standardized mean difference = 0.558), median left ventricular end-systolic diameter was 61 mm versus 60 mm (56–66 mm vs. 57–69 mm; standardized mean difference = 0.255), and tricuspid annular plane systolic excursion was 13 mm versus 14 mm (11–16 mm vs. 11–17 mm; standardized mean difference = 0.186). These findings indicate that baseline left ventricular geometry and right ventricular function were similar across groups, although residual confounding cannot be excluded.

Postoperative echocardiographic assessment confirmed persistent comparability between groups. Median left ventricular end-diastolic diameter was 68 mm in the LVT cohort versus 65 mm in controls (*p* = 0.103), median left ventricular end-systolic diameter was 59 mm versus 53 mm (*p* = 0.096), and tricuspid annular plane systolic excursion was identical at 14 mm (12–16 mm; *p* = 0.592). These results suggest preserved left ventricular size, systolic function, and right ventricular performance postoperatively, irrespective of preoperative thrombus status. Improved left ventricular function during durable LVAD support has been associated with lower, rather than increased, risk of device thrombosis [21]. Our findings suggest that when preoperative LVT is surgically addressed and LVAD therapy is optimized, structural cardiac parameters remain stable, though this observation should be interpreted with caution given the limited sample size.

Aortic valve opening patterns did not differ significantly between groups (*p* = 0.289); continuous or intermittent opening was observed in 61.9% of LVT patients and 81% of controls, while persistent closure was noted in 38.1% and 19%, respectively. These patterns are an important echocardiographic parameter during LVAD support, as they influence aortic root flow dynamics and may impact long-term outcomes. Intermittent aortic valve opening has been associated with measurable hemodynamic benefits, including enhanced platelet washout, reduced stasis, and decreased overall device thrombogenicity. Strategies permitting partial or low-frequency intermittent aortic valve opening may improve biocompatibility and reduce the risk of thrombotic complications [22]. In our cohort, the lack of significant differences in aortic valve opening patterns suggests that in patients with preoperative left ventricular thrombosis who underwent surgical thrombectomy, postoperative hemodynamic unloading and valve function appeared to be preserved at levels comparable to patients without thrombus. These findings suggest that careful surgical management of LVT, combined with optimized LVAD support, may not adversely affect aortic valve dynamics. No statistically significant differences were observed in the adverse outcomes associated with permanent valve closure between the two groups.

Several limitations of the present study should be acknowledged. First, this study has a retrospective, single-center design, which inherently introduces the potential for selection bias and limits the generalizability of the findings to broader LVAD populations. Second, the overall sample size was relatively small, particularly after propensity score matching, resulting in a limited number of outcome events. Consequently, the study may have been underpowered to detect small but clinically relevant differences between groups. Third, despite the use of propensity score matching to reduce baseline differences, residual imbalance remained for certain variables, particularly sex and hypertension, with standardized mean differences exceeding commonly accepted thresholds (>0.20). This residual imbalance may introduce some residual confounding and could affect the internal validity of the comparisons. The variables included in the propensity model were selected based on clinical relevance and literature-reported risk factors for thromboembolic events in LVAD patients. Although complete covariate balance was not achievable due to the relatively small sample size, the overall trends in postoperative outcomes suggest that these imbalances are unlikely to substantially alter the study’s main findings.

Fourth, as precise timing data for postoperative events were not available, time-to-event analyses such as Kaplan–Meier analysis could not be performed. The current analyses are exploratory in nature, and no formal adjustment for multiple comparisons was applied.

Furthermore, the wide confidence intervals observed in several effect size estimates reflect substantial statistical uncertainty, which is mainly attributable to the small sample size and low number of events. Therefore, these findings should be interpreted with caution.

In addition, precise data regarding the total duration of anticoagulation therapy were not available, except for patients diagnosed with atrial fibrillation preoperatively, as treatment was initiated upon hospital admission and its duration varied depending on the timing of surgery. This limits a more detailed assessment of the potential impact of preoperative anticoagulation on postoperative outcomes.

Finally, the inclusion of different LVAD types introduces a degree of heterogeneity within the study population. Although device type may influence thrombotic and neurological outcomes, it was not the primary focus of this study and was therefore not specifically analyzed.

## 5. Conclusions

In this propensity score-matched cohort of patients undergoing durable LVAD implantation with surgical thrombectomy, the presence of preoperative left ventricular thrombus (LVT) was not associated with a statistically significant increase in the risk of postoperative stroke, pump thrombosis, infectious complications, ventricular arrhythmias, or adverse echocardiographic outcomes during early and mid-term follow-up; however, clinically meaningful differences cannot be excluded given the limited sample size and wide confidence intervals. Clinical outcomes, including duration of device support and postoperative ventricular parameters, were comparable between patients with and without preoperative LVT.

These findings suggest that, when meticulous intraoperative evaluation and complete surgical thrombectomy are performed, pre-existing left ventricular thrombus may not be associated with worse clinical outcomes after LVAD implantation.

However, these results should be interpreted with caution given the residual baseline imbalances and the exploratory nature of this analysis. Further prospective, multicenter studies with larger patient populations are needed to confirm these findings and to better define the long-term clinical impact of preoperative LVT in patients receiving durable mechanical circulatory support.

## Figures and Tables

**Table 1:** Baseline demographic, clinical, and echocardiographic characteristics of patients with and without left ventricular thrombosis after propensity score matching *.

Variables	Control (*n* = 21)	Left Ventricular Thrombus (*n* = 21)	SMD
	*n* (%) or Median (IQR)	*n* (%) or Median (IQR)	
Gender			−0.649
Male	21 (100)	17 (81)	
Female	0 (0)	4 (19.0)	
Age (years)	56 (47–62)	57 (49–62)	0.175
BMI (kg/m^2^)	25 (23–26.7)	23.4 (21.2–24.8)	−0.296
**Type of Cardiomyopathy**			
ICMP	12 (57.1)	11 (52.4)	−0.096
DCMP	8 (38.1)	8 (38.1)	0.000
ARVD	0 (0)	1 (4.8)	0.312
RCMP	0 (0)	0 (0)	0.000
Hypertension	7 (33.3)	13 (61.9)	0.572
Diabetes Mellitus	4 (19.0)	7 (33.3)	0.325
Preoperative AF	4 (19.0)	6 (28.6)	0.224
Carotid Artery Disease	0 (0)	0 (0)	0.000
Preoperative CVE	1 (4.8)	0 (0)	−0.312
Preoperative LVEDD (mm)	67 (60–70)	67 (65–71)	0.558
Preoperative LVESD (mm)	61 (56–66)	60 (57–69)	0.255
Preoperative TAPSE (mm)	13 (11–16)	14 (11–17)	0.186
Preoperative LDL (mg/dL)	75 (60–103)	83 (69–98)	0.141
**LVAD Brand**			
HM2	0 (0)	2 (9.5)	0.447
HM3	18 (85.7)	15 (71.4)	−0.348
HVAD	3 (14.3)	4 (19.0)	0.130
Mechanical MVR	0 (0)	1 (4.8)	0.312

Data are presented as n (%) or median (interquartile range, IQR). Post-matching covariate balance was assessed using standardized mean differences (SMDs). An absolute SMD value greater than 0.20 was considered indicative of residual imbalance. Abbreviations: BMI, body mass index; ICMP, ischemic cardiomyopathy; DCMP, dilated cardiomyopathy; ARVD, arrhythmogenic right ventricular dysplasia; RCMP, restrictive cardiomyopathy; HT, hypertension; DM, diabetes mellitus; AF, atrial fibrillation; CVE, cerebrovascular event; LDL, low-density lipoprotein; LVEDD, left ventricular end-diastolic diameter; LVESD, left ventricular end-systolic diameter; TAPSE, tricuspid annular plane systolic excursion; HM2, HeartMate 2 (Abbott Inc., Abbott Park, IL, USA); HM3, HeartMate 3 (Abbott Inc., Abbott Park, IL, USA); HVAD, HeartWare HVAD (Medtronic, Inc., Minneapolis, MN, USA); MVR, mitral valve replacement; SMDs, standardized mean differences; PSM, propensity score matching; IQR, interquartile range. * Following propensity score matching, 21 patients with left ventricular thrombus and 21 control patients were matched, resulting in 21 matched pairs. After matching, covariate balance between groups was assessed using the standardized mean difference (SMD). In propensity score matching (PSM) analyses, SMD is preferred over *p*-values for assessing covariate balance, as *p*-values are influenced by sample size and may fail to detect clinically meaningful differences in small samples. In contrast, SMD reflects the magnitude of differences between groups independently of sample size. An absolute SMD value of <0.10 is considered indicative of good balance, 0.10–0.20 indicates acceptable balance, and >0.20 suggests residual imbalance. In this study, although balance improved for most baseline covariates included in the matching model, moderate to high SMD values persisted in some variables. In particular, sex, hypertension, preoperative LVEDD, and certain LVAD subgroups showed absolute SMD values exceeding 0.20. This finding may be related to the limited number of variables included in the matching model and the higher proportion of male patients in the control group. Covariate balance after propensity score matching was evaluated using standardized mean difference (SMD) rather than *p*-values, which are sensitive to sample size.

**Table 2:** Comparison of postoperative clinical outcomes in a propensity-score-matched cohort.

Variables	Control (*n* = 21)	Left Ventricular Thrombus (*n* = 21)	*p*-Value	Effect Sizes †
	*n* (%) or Median (IQR)	*n* (%) or Median (IQR)		
Postoperative Ischemic Stroke	1 (4.8)	1 (4.8)	1.000	1.00 (0.06–15.9)
Postoperative Hemorrhagic Stroke	0 (0)	1 (4.8)	1.000	NE
Postoperative Pump Thrombosis	1 (4.8)	2 (9.5)	1.000	2.00 (0.18–22.1)
Driveline Infection	12 (57.1)	7 (33.3)	0.227	0.44 (0.13–1.54)
Bloodstream Infection	6 (28.6)	4 (19.0)	0.688	0.60 (0.14–2.59)
Postoperative VF, VT	5 (23.8)	4 (19.0)	1.000	0.67 (0.11–4.02)
LA Thrombus	0 (0)	1 (4.8)	1.000	NE
LVAD Duration (days)	821 (709–1028)	1003 (427–1935)	0.892	−167 (−3033, NE)
Postoperative LVEDD (mm)	65 (53–69)	68 (63–72)	0.103	8 (−35, NE)
Postoperative LVESD (mm)	53 (46–65)	59 (55–67)	0.096	9 (−34, NE)
Postoperative TAPSE (mm)	14 (12–16)	14 (12–16)	0.592	0 (−6, NE)
Aortic Valve Opening			0.289	2.33 (0.61–8.97)
Opening/Intermittently Opening	17 (81.0)	13 (61.9)		
Not Opening At All	4 (19.0)	8 (38.1)		
Amiodarone	7 (33.3)	11 (52.4)	0.289	2.33 (0.61–8.97)

Data are presented as n (%) or median (interquartile range, IQR). Categorical variables were compared using the McNemar test for paired samples; continuous variables were analyzed using the Wilcoxon signed-rank test. A *p*-value < 0.05 was considered statistically significant. Effect sizes are reported as matched odds ratios (95% confidence intervals) for categorical variables and as paired median differences (patient − control, percentile range) for continuous variables. NE: Not estimable due to small sample size (upper confidence interval limit could not be calculated). Abbreviations: VF, ventricular fibrillation; VT, ventricular tachycardia; LA, left atrium; LVEDD, left ventricular end-diastolic diameter; LVESD, left ventricular end-systolic diameter; TAPSE, tricuspid annular plane systolic excursion; LVT, left ventricular thrombus; LVAD, left ventricular assist device; IQR, interquartile range; NE, not estimable. † When postoperative clinical outcomes were compared in the matched cohort, no statistically significant differences were observed between patients with left ventricular thrombus and the control group in terms of the evaluated complications, device-related events, or postoperative echocardiographic parameters (all *p* > 0.05). Categorical variables were analyzed using McNemar’s test for matched samples, while continuous variables were assessed using the Wilcoxon signed-rank test. In effect size analyses, the matched odds ratio for postoperative pump thrombosis was 2.00 (95% confidence interval: 0.18–22.1), and for aortic valve opening and amiodarone use, the matched odds ratio was 2.33 (95% confidence interval: 0.61–8.97). For continuous variables, paired median differences were −167 days for device support duration, +8 mm for postoperative LVEDD, +9 mm for postoperative LVESD, and 0 for postoperative TAPSE.

## Data Availability

The data presented in this study are available on request from the corresponding author. Restrictions apply due to privacy, ethical, and institutional regulations.

## Abbreviations

LVT	left ventricular thrombus
LVAD	left ventricular assist device
HF	Advanced heart failure
INTERMACS	Interagency Registry for Mechanically Assisted Circulatory Support
CMR	cardiac magnetic resonance imaging
BMI	body mass index
LDL	low-density lipoprotein
LVEDD	left ventricular end-diastolic diameter
LVESD	left ventricular end-systolic diameter
TAPSE	tricuspid annular plane systolic excursion
PSM	propensity score matching
SMD	standardized mean differences
ICMP	ischemic cardiomyopathy
DCMP	dilated cardiomyopathy
ARVD	arrhythmogenic right ventricular dysplasia
RCMP	restrictive cardiomyopathy
HT	hypertension
DM	diabetes mellitus
AF	atrial fibrillation
CVE	cerebrovascular events
HM2	Heartmate 2 (Abbott Inc., Abbott Park, IL, USA)
HM3	Heartmate 3 (Abbott Inc., Abbott Park, IL, USA)
HW	HeartWare (HVAD) (Medtronic, Inc., Minneapolis, MN, USA)
VF	Ventricular fibrillation
VT	Ventricular tachycardia
LA	Left atrium

## References

[1] Kirklin J.K., Pagani F.D., Kormos R.L., Stevenson L.W., Blume E.D., Myers S.L., Miller M.A., Baldwin J.T., Young J.B., Naftel D.C. (2017). Eighth annual INTERMACS report: Special focus on framing the impact of adverse events. J. Heart Lung Transplant..

[2] Mehra M.R., Uriel N., Naka Y., Cleveland J.C., Yuzefpolskaya M., Salerno C.T., Walsh M.N., Milano C.A., Patel C.B., Hutchins S.W. (2019). A fully magnetically levitated left ventricular assist device—Final report. N. Engl. J. Med..

[3] Molina E.J., Shah P., Kiernan M.S., Cornwell W.K., Copeland H., Takeda K., Fernandez F.G., Badhwar V., Habib R.H., Jacobs J.P. (2021). The Society of Thoracic Surgeons Intermacs 2020 annual report. Ann. Thorac. Surg..

[4] Bravo C.A., Navarro A.G., Dhaliwal K.K., Khorsandi M., Keenan J.E., Mudigonda P., O’Brien K.D., Mahr C. (2022). Right heart failure after left ventricular assist device: From mechanisms to treatments. Front. Cardiovasc. Med..

[5] McCarthy C.P., Murphy S., Venkateswaran R.V., Singh A., Chang L.L., Joice M.G., Rivero J.M., Vaduganathan M., Januzzi J.L., Bhatt D.L. (2019). Left ventricular thrombus: Contemporary etiologies, treatment strategies, and outcomes. J. Am. Coll. Cardiol..

[6] Delewi R., Zijlstra F., Piek J.J. (2012). Left ventricular thrombus formation after acute myocardial infarction. Heart.

[7] Weinsaft J.W., Kim H.W., Shah D.J., Klem I., Crowley A.L., Brosnan R., James O.G., Patel M.R., Heitner J., Parker M. (2008). Detection of left ventricular thrombus by delayed-enhancement cardiac magnetic resonance imaging. J. Am. Coll. Cardiol..

[8] Chaosuwannakit N., Makarawate P. (2021). Left ventricular thrombi: Insights from cardiac magnetic resonance imaging. Tomography.

[9] Bravo C.A., Fried J.A., Willey J.Z., Javaid A., Mondellini G.M., Braghieri L., Lumish H., Topkara V.K., Kaku Y., Witer L. (2021). Presence of intracardiac thrombus at the time of left ventricular assist device implantation is associated with an increased risk of stroke and death. J. Card. Fail..

[10] Fried J.A., Lumish H., Zuver A.M., Garan A.R., Topkara V.K., Cagliostro B., Parkis G., Cevasco M., Pineda M., Mondellini G. (2019). Presence of left atrial or left ventricular thrombus at the time of continuous-flow LVAD implantation is associated with increased postoperative risk of stroke or death. J. Heart Lung Transplant..

[11] Acharya D., Loyaga-Rendon R., Morgan C.J., Sands K.A., Pamboukian S.V., Rajapreyar I., Holman W.L., Kirklin J.K., Tallaj J.A. (2017). INTERMACS analysis of stroke during support with continuous-flow left ventricular assist devices: Risk factors and outcomes. JACC Heart Fail..

[12] Li S., Beckman J.A., Cheng R., Ibeh C., Creutzfeldt C.J., Bjelkengren J., Herrington J., Stempien-Otero A., Lin S., Levy W.C. (2020). Comparison of neurologic event rates among HeartMate II, HeartMate 3, and HVAD left ventricular assist devices. ASAIO J..

[13] Starling R.C., Moazami N., Silvestry S.C., Ewald G., Rogers J.G., Milano C.A., Rame J.E., Acker M.A., Blackstone E.H., Ehrlinger J. (2014). Unexpected abrupt increase in left ventricular assist device thrombosis. N. Engl. J. Med..

[14] Dogan G., Mariani S., Hanke J.S., Deniz E., Merzah A., Li T., Haverich A., Schmitto J.D. (2021). Left ventricular assist device implantation in patients with left ventricular thrombus. Artif. Organs.

[15] Teuteberg J.J., Cleveland J.C., Cowger J., Higgins R.S., Goldstein D.J., Keebler M., Kirklin J.K., Myers S.L., Salerno C.T., Stehlik J. (2020). The Society of Thoracic Surgeons Intermacs 2019 annual report: The changing landscape of devices and indications. Ann. Thorac. Surg..

[16] Stulak J.M., Deo S., Schirger J., Aaronson K.D., Park S.J., Joyce L.D., Daly R.C., Pagani F.D. (2013). Preoperative atrial fibrillation increases risk of thromboembolic events after left ventricular assist device implantation. Ann. Thorac. Surg..

[17] Hindricks G., Potpara T., Dagres N., Arbelo E., Bax J.J., Blomström-Lundqvist C., Boriani G., Castella M., Dan G.A., Dilaveris P.E. (2021). 2020 ESC Guidelines for the diagnosis and management of atrial fibrillation developed in collaboration with the European Association for Cardio-Thoracic Surgery (EACTS): The Task Force for the diagnosis and management of atrial fibrillation of the European Society of Cardiology (ESC) Developed with the special contribution of the European Heart Rhythm Association (EHRA) of the ESC. Eur. Heart J..

[18] Garan A.R., Yuzefpolskaya M., Colombo P.C., Morrow J.P., Te-Frey R., Dano D., Takayama H., Naka Y., Garan H., Jorde U.P. (2013). Ventricular arrhythmias and implantable cardioverter-defibrillator therapy in patients with continuous-flow left ventricular assist devices: Need for primary prevention?. J. Am. Coll. Cardiol..

[19] Schmitto J.D., Shaw S., Garbade J., Gustafsson F., Morshuis M., Zimpfer D., Lavee J., Pya Y., Berchtold-Herz M., Wang A. (2024). Fully magnetically centrifugal left ventricular assist device and long-term outcomes: The ELEVATE registry. Eur. Heart J..

[20] Kyriakopoulos C.P., Horne B.D., Sideris K., Taleb I., Griffin R.J., Sheffield E., Alharethi R., Hanff T.C., Stehlik J., Selzman C.H. (2023). Left ventricular functional improvement appears to contribute to lower rates of device thrombosis in patients on durable mechanical circulatory support. J. Heart Lung Transplant..

[21] Mahr C., Chivukula V., McGah P., Prisco A.R., Beckman J.A., Mokadam N.A., Aliseda A. (2017). Intermittent aortic valve opening and risk of thrombosis in VAD patients. ASAIO J..

[22] Malone G., Abdelsayed G., Bligh F., Al Qattan F., Syed S., Varatharajullu P., Msellati A., Mwipatayi D., Azhar M., Malone A. (2022). Advancements in left ventricular assist devices to prevent pump thrombosis and blood coagulopathy. J. Anat..

